# A Randomized, Double-Blind, Placebo-Controlled Single-Ascending-Dose Study to Identify a Subperceptual Dose of Psilocybin in Healthy Adults

**DOI:** 10.3390/ph19091496

**Published:** 2026-09-21

**Authors:** Naama Levy-Cooperman, Edward Sellers, Paul Glue, Isabella Szeto, David Brown, Jamie Jarecki-Smith, William J. Tyler, Michael B. McDonnell

**Affiliations:** 1 Altreos Research Partners, Inc., Toronto, ON M6P 1C6, Canada; 2 Diamond Therapeutics, Inc., Toronto, ON M5X 1B8, Canada; 3 Biopharma Services, Inc., Toronto, ON M9L 3A2, Canada; 4 Thermo Fisher Scientific, Mississauga, ON L5N 7K9, Canada; 5 Diamond Therapeutics USA, Inc., Birmingham, AL 35294, USA

**Keywords:** psilocybin, psilocin, pharmacodynamics, pharmacokinetics, generalized anxiety disorder

## Abstract

**Background/Objectives:** Psilocybin shows therapeutic promise for several psychiatric disorders, but the acute perceptual and cognitive alterations produced by conventional doses (10–25 mg) require in-clinic supervision, which limits scalability. Whether the therapeutically relevant pharmacology of psilocybin can be safely separated from its hallucinogenic activity remains unresolved. To address this gap, we conducted a Phase 1, randomized, double-blind, placebo-controlled, single-ascending-dose study to characterize the safety, pharmacokinetics, and pharmacodynamics of low doses of psilocybin. **Methods:** Fifty-six healthy adults received a single oral dose of psilocybin (0.5, 1.0, 1.5, 2.5, 3.5 or 4.0 mg) or matching placebo across seven sequential cohorts, with each dose escalation reviewed by a Drug Safety Review Committee. All participants completed the study with no serious adverse events or discontinuations. **Results:** Treatment-emergent adverse events were comparable to placebo and most prominently arose as somnolence. Plasma psilocin appeared rapidly with a median time to maximum concentration <1 h with dose-proportional exposure and a short terminal half-life. Subjective drug effects were dose-related and became distinguishable from placebo at doses ≥2.5 mg. Peak subjective ratings increased with dose, while altered-state scores remained low and cognitive changes did not differ from placebo. Psychophysiological engagement was confirmed by a clear dose-dependent pupillary dilation, while cognitive performance (attention, vigilance, working memory, impulse control) showed no dose-dependent decrement, and state anxiety did not increase at any dose. **Conclusions:** These findings suggest that low-dose psilocybin may produce perceptible pharmacological effects without significant perceptual alterations or cognitive impairment. The results also offer preliminary support for considering controlled outpatient Phase 2 studies assessing the safety and feasibility of repeated, self-administered low-dose psilocybin. Retrospective ClinicalTrials.gov Registration on 17 July 2026 #NCT07710027.

## 1. Introduction

Psilocybin is a naturally occurring tryptamine alkaloid produced by numerous species of *Psilocybe* mushrooms. After oral administration, the prodrug is rapidly dephosphorylated to its active metabolite, psilocin, an agonist at multiple serotonin receptor subtypes, with effects on perception and cognition attributed primarily to agonism at the 5-HT2A receptor [1,2,3]. Over the past two decades, a growing body of research has indicated that classic tryptamine psychedelics may produce rapid and durable reductions in symptoms of depression, anxiety, obsessive–compulsive disorder, and substance use disorders [4,5,6]. Anxiety disorders, and generalized anxiety disorder (GAD) in particular, are among the most common psychiatric conditions, yet they remain frequently underdiagnosed and undertreated [7,8,9]. The persistence of this substantial unmet need represents a public health crisis further motivating interest in the development of treatments with novel mechanisms, including serotonergic psychedelics.

Most studies investigating the therapeutic benefits of psilocybin to date have used moderate-to-high doses (10 to 25 mg), which reliably produces intense, hours-long altered states of consciousness [10,11]. Due to these effects, including significant perceptual distortions, altered sense of self, and heightened sympathetic activity, continuous in-clinic supervision is required at these doses creating a therapeutic bottleneck. Thus, the very feature that may drive the beneficial psychological effects of psilocybin also prevent it from scaling as a therapy. Whether therapeutic activity and intensity of perceptual alterations induced by psilocybin are mechanistically inseparable, or merely correlated at the doses studied thus far, remains an open and clinically consequential question. Several lines of evidence suggest they may, in fact, be separable and motivates direct empirical testing.

Real-world patterns show that people are already self-selecting a low-dose regime. An estimated 11 million adults in the USA used low, sub-hallucinogenic doses of psilocybin in 2025 [12], primarily to manage anxiety and depressive symptoms, and naturalistic survey data associate this pattern of use with lower anxiety and depression compared to non-user controls [13,14,15]. These data are confounded by expectancy and self-selection, but the scale and consistency of the signals argue against dismissing them outright. Early controlled clinical data point the same direction. In patients with advanced illness and psychological distress, oral psilocybin titrated to 1–3 mg produced no psychedelic experiences yet was associated with meaningful improvements in depression, anxiety, and demoralization, with the clearest benefits clustering at the 3 mg dose [16]. Additional preclinical work provides a plausible mechanism for this dissociation.

Low, sub-hallucinogenic doses of psilocybin and ketamine improve attention and motivation in animal models without producing hallucinogenic behavioral markers [17], and repeated low-dose psilocybin increases synaptic density and stress resilience in rodents [18]. These effects may be mediated through BDNF/TrkB signaling independent of the surface 5-HT2A activity that drives classical hallucinations [19,20,21]. Pharmacokinetic and receptor-occupancy data give this rationale an additional quantitative anchor. Subjective effects emerge at plasma psilocin concentrations of roughly 4–6 ng/mL [11], corresponding to a PET-derived EC_50_ of 4.5 ng/mL [3], and perceptual effects appear minimal below ~15% cortical 5-HT2A occupancy [22]. Together, these data define a plausible pharmacological window where central serotonergic engagement could occur without crossing the threshold for distorted perception, but this window has never been systematically mapped in humans under controlled conditions.

Collectively, these observations warrant systematic and controlled studies designed to characterize perceptual and hallucinogenic thresholds of low doses of psilocybin in humans. Naturalistic and open-label data cannot establish a defensible, quantitative dose threshold, which requires a randomized, blinded, placebo-controlled dose-escalation design. We therefore conducted this randomized, double-blind, placebo-controlled Phase 1 single-ascending-dose study to characterize the safety, pharmacokinetics, and pharmacodynamics of low-dose psilocybin (0.5–4 mg) in healthy adults, with the primary objective of identifying the highest dose that is pharmacologically perceptible and therefore plausibly therapeutically active without producing hallucinatory activity, cognitive impairment, or intense altered states that currently require in-clinic supervision. Our primary aim in this study was to identify this threshold as an empirically derived starting point for efficacy trials in GAD and other neuropsychiatric conditions to evaluate a scalable, self-administered outpatient treatment model.

## 2. Results

### 2.1. Participant Characteristics

**Figure 1:**
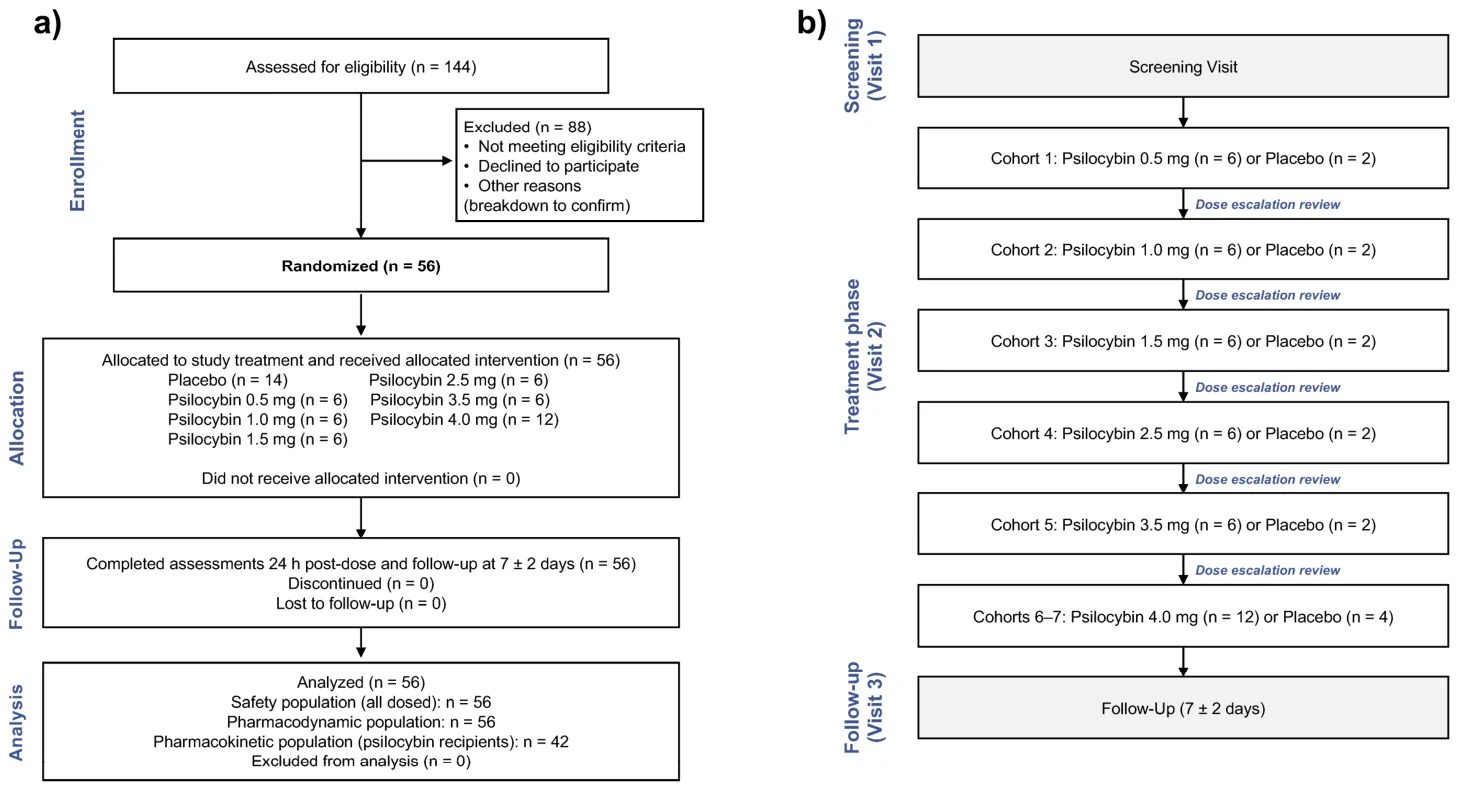
Clinical trial design and dose escalation flow. (**a**) A CONSORT diagram is illustrated for the study. We assessed 144 individuals for eligibility; 88 were excluded, and 56 were randomized. All randomized participants received an allocated treatment, completed the study, and were analyzed (safety n = 56; pharmacodynamic n = 56; pharmacokinetic n = 42), with no losses or discontinuations. (**b**) The study design and dose-escalation scheme are illustrated across the three study visits (screening; inpatient treatment phase; follow-up at 7 ± 2 days). Seven sequential cohorts of 8 participants each randomized on a 3:1 ratio (6 psilocybin, 2 placebo) received single oral doses of 0.5, 1.0, 1.5, 2.5, 3.5, and 4.0 mg. We repeated the 4.0 mg dose in two cohorts (n = 12 each), with a Drug Safety Review Committee reviewing blinded data in between each cohort.

Of 144 individuals screened, 56 were randomized and dosed, as follows: 42 received psilocybin (n = 6 each at 0.5, 1.0, 1.5, 2.5 and 3.5 mg, and n = 12 at 4.0 mg) and 14 received placebo (2 per cohort). All 56 participants received their assigned dose and completed the study. No participants discontinued or were lost to follow-up, and all were included in the safety and pharmacodynamic analyses (Figure 1a). Participants were evenly divided by sex (28 men, 28 women); the largest racial group was White (26/56; 46.4%) and most were not Hispanic or Latino (45/56; 80.4%). The mean (standard deviation, SD) age was 37.5 (10.2) years and mean (SD) body mass index 26.28 (3.86) kg/m^2^. Baseline characteristics and demographics are shown in Table 1.

### 2.2. Low-Dose Psilocybin Pharmacokinetics Following Oral Administration

**Figure 2:**
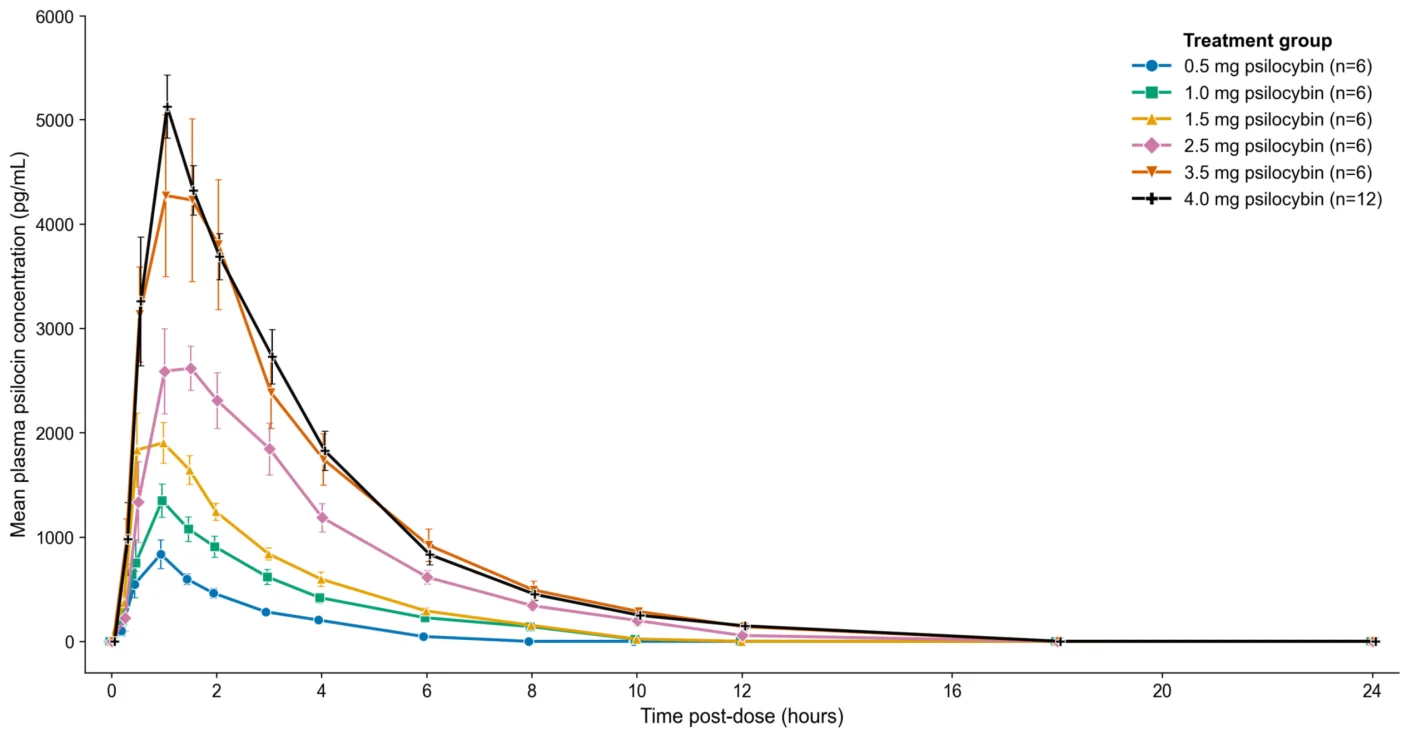
Pharmacokinetic profile of orally administered low-dose psilocybin. Line plots illustrate the mean ± SEM plasma psilocin concentrations over time by psilocybin dose.

Following single oral doses of 0.5 to 4.0 mg, psilocin appeared rapidly in plasma and was quantifiable in most participants by 0.25 to 0.5 h following dose administration (Figure 2). Peak plasma concentrations were reached at a median of approximately 1 h and increased in a clear dose-ordered manner. The mean ± SEM peak concentration on the concentration–time profile rose from 835 ± 138 pg/mL (0.835 ± 0.138 ng/mL) at 0.5 mg to 1349 ± 161 pg/mL (1.349 ± 0.161 ng/mL) at 1.0 mg, 1902 ± 195 pg/mL (1.902 ± 0.195 ng/mL) at 1.5 mg, 2618 ± 212 pg/mL (2.618 ± 0.212 ng/mL) at 2.5 mg, 4272 ± 777 pg/mL (4.272 ± 0.777 ng/mL) at 3.5 mg and 5127 ± 303 pg/mL (5.127 ± 0.303 ng/mL) at 4.0 mg. As illustrated in Figure 2, the median time to maximum concentration (T_max_) was comparable across doses, ranging from approximately 0.7 to 1.4 h, with the latest peak at 3.5 mg (median 1.4 h). Concentrations then declined in a biphasic manner. This was characterized by an initial rapid phase followed by a slower terminal phase. Most participants had fallen below the limit of quantitation (<120 pg/mL or 0.120 ng/mL) by 10 to 18 h. The median terminal elimination half-life (t½) was short and essentially dose-independent, increasing only modestly from approximately 1.6 h at 0.5 mg to 2.3 to 2.5 h at doses of 1.0–4.0 mg. Total exposure increased with dose by non-compartmental analysis; mean ± SD C_max_ rose from 905 ± 363 pg/mL (0.905 ± 0.363 ng/mL) at 0.5 mg to 5360 ± 1153 pg/mL (5.360 ± 1.153 ng/mL) at 4.0 mg, and mean AUC_last_ from 1765 ± 516 to 17,181 ± 3767 pg·h/mL (1.765 ± 0.16 to 17.181 ± 3.767 ng·h/mL) over the same range. Across the 0.5 to 4.0 mg range AUC increased in a dose-proportional manner, whereas C_max_ increased slightly less than dose-proportionally.

### 2.3. Subjective Pharmacodynamic Effects of Orally Administered Low-Dose Psilocybin

**Figure 3:**
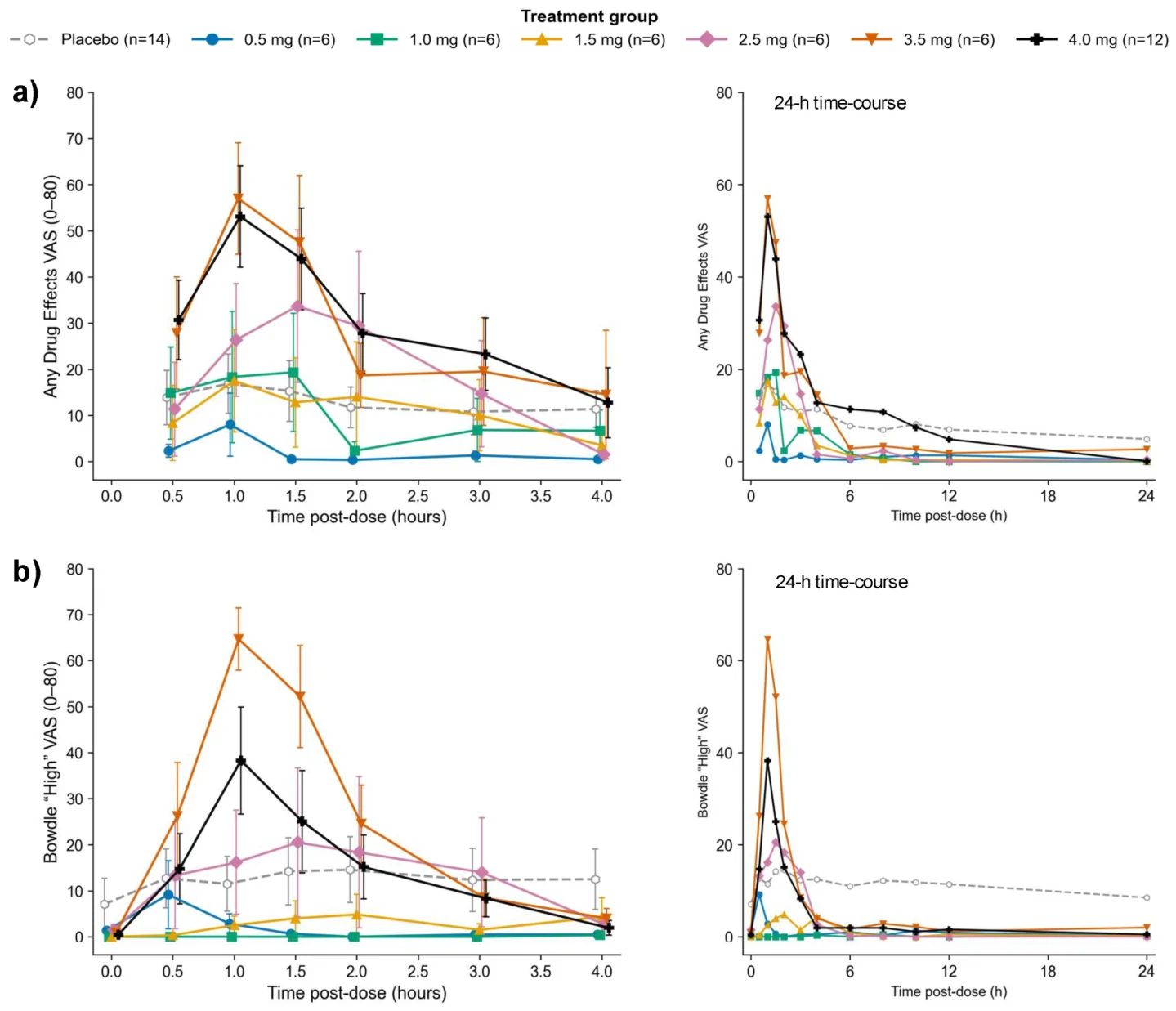
Dose responses for subjective pharmacodynamic experiences across time. The line plots illustrate subjective drug-effect time courses by dose as cohort mean ± SEM for the first 4 h (**left**) and 24 h (**right**) following psilocybin or placebo administration. Data from the (**a**) Any Drug Effects VAS (“At this moment, I feel any drug effects”) and (**b**) Bowdle “High” VAS (“I felt high”) survey items, each scored 0–100, for placebo and the six psilocybin doses are illustrated.

On the Any Drug Effects VAS, scores increased rapidly after dosing and peaked approximately 1 h following dose administration (Figure 3a). We observed that peak scores were higher than placebo for psilocybin doses ≥2.5 mg and similar or lower than placebo for doses <2.5 mg. The most robust responses occurred at the 3.5 and 4.0 mg doses, although the within-group variability was greatest at these doses. A parallel pattern was seen for the Bowdle “I felt high” rating, which increased from 0.5 h post-dose and peaked between 1 and 1.5 h at doses ≥2.5 mg, again with the greatest increases at the two highest doses (Figure 3b). Scores at 2.5 mg were like those observed for placebo, and from 0.5 to 1.5 mg remained essentially flat. Despite the increased subjective perceptual scores at the two highest doses (3.5 and 4.0 mg), these changes were short lived (Figure 3b) and not associated with any impairments in cognitive function or performance (see Section 2.6).

**Figure 4:**
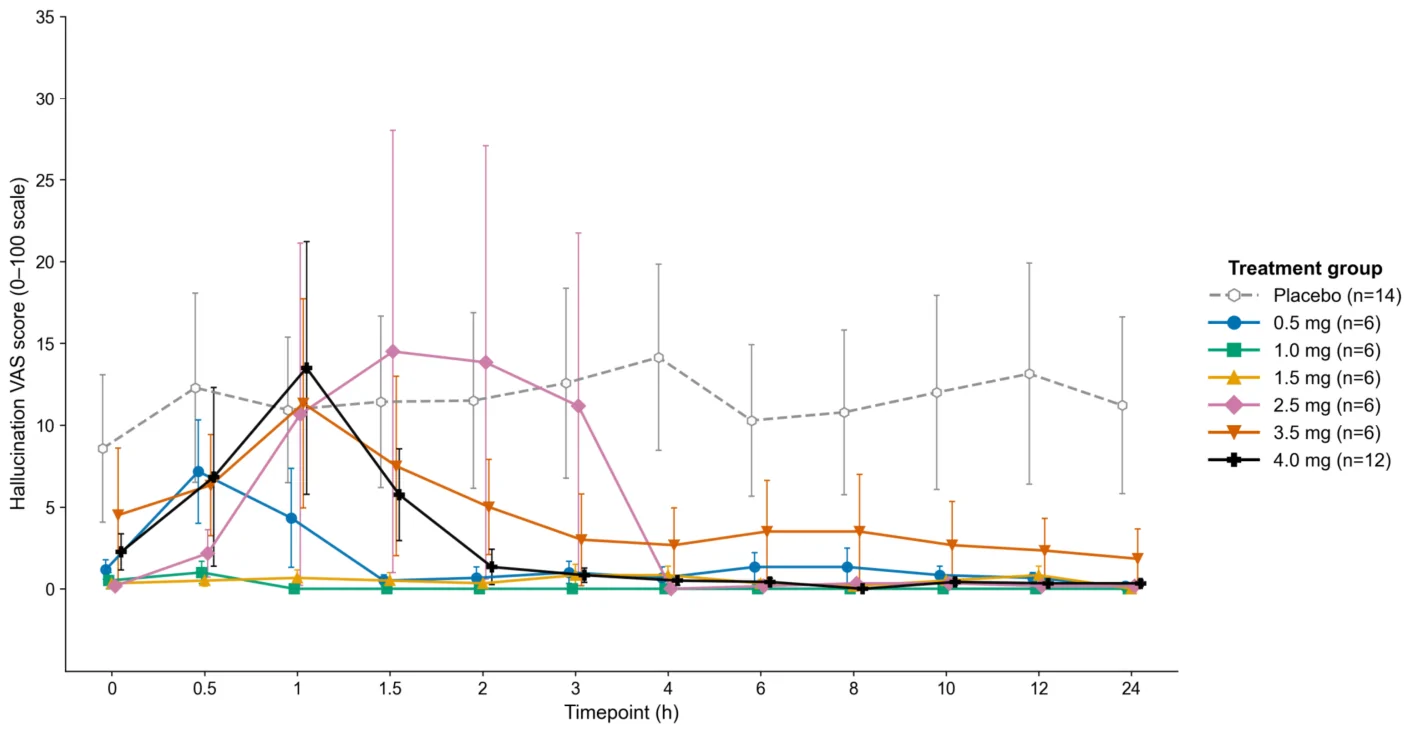
Subjective perceptual changes across low doses of psilocybin by time. The line plots illustrate subjective hallucination visual analog scores (VAS) on a scale of 0 (low) to high (100) truncated at 35 for each dose and placebo across the 24 h time period following treatment. There was no significant correlation between dose and the intensity of subjective experiences across the low psilocybin doses investigated, and placebo produced results indistinguishable from active drug effects. Data are illustrated as cohort mean ± SEM.

Scores reflecting overt perceptual disturbance remained low and showed no dose dependence. Peak Hallucination VAS scores did not increase with dose (Spearman ρ = −0.05, *p* = 0.69), with the highest mean peak occurring in the placebo group (Figure 4). Individual scores exceeded 20 of 100 in 4 of 14 placebo recipients, versus 1 of 6 at 2.5 mg, 2 of 6 at 3.5 mg, and 3 of 12 at 4.0 mg, indicating that the recognizable drug effects were not attributable to altered consciousness or distorted perceptions (Figure 4). Likewise, the Bowdle items indexing perceptual distortions including altered perception of colors, sounds, and one’s body showed no dose-related increase (all ρ ≈ −0.05, *p* > 0.6), with mean peak ratings generally below 20 of 100. The Bowdle “Anxious” item showed only a modest, non-significant tendency to increase with dose (ρ = 0.23, *p* = 0.09), with the highest mean peak at 4.0 mg. On the Agitation/Relaxation VAS, participants were relatively relaxed before and after dosing across all groups. On the Alertness/Drowsiness VAS, scores were generally consistent with an alert state and revealed no consistent dose-related pattern. Bond-Lader affective dimensions changed minimally, although 4.0 mg was associated with a greater decrease in the Alertness dimension than other groups.

### 2.4. Effects of Low-Dose Psilocybin on Perception and Sustained Attention

**Figure 5:**
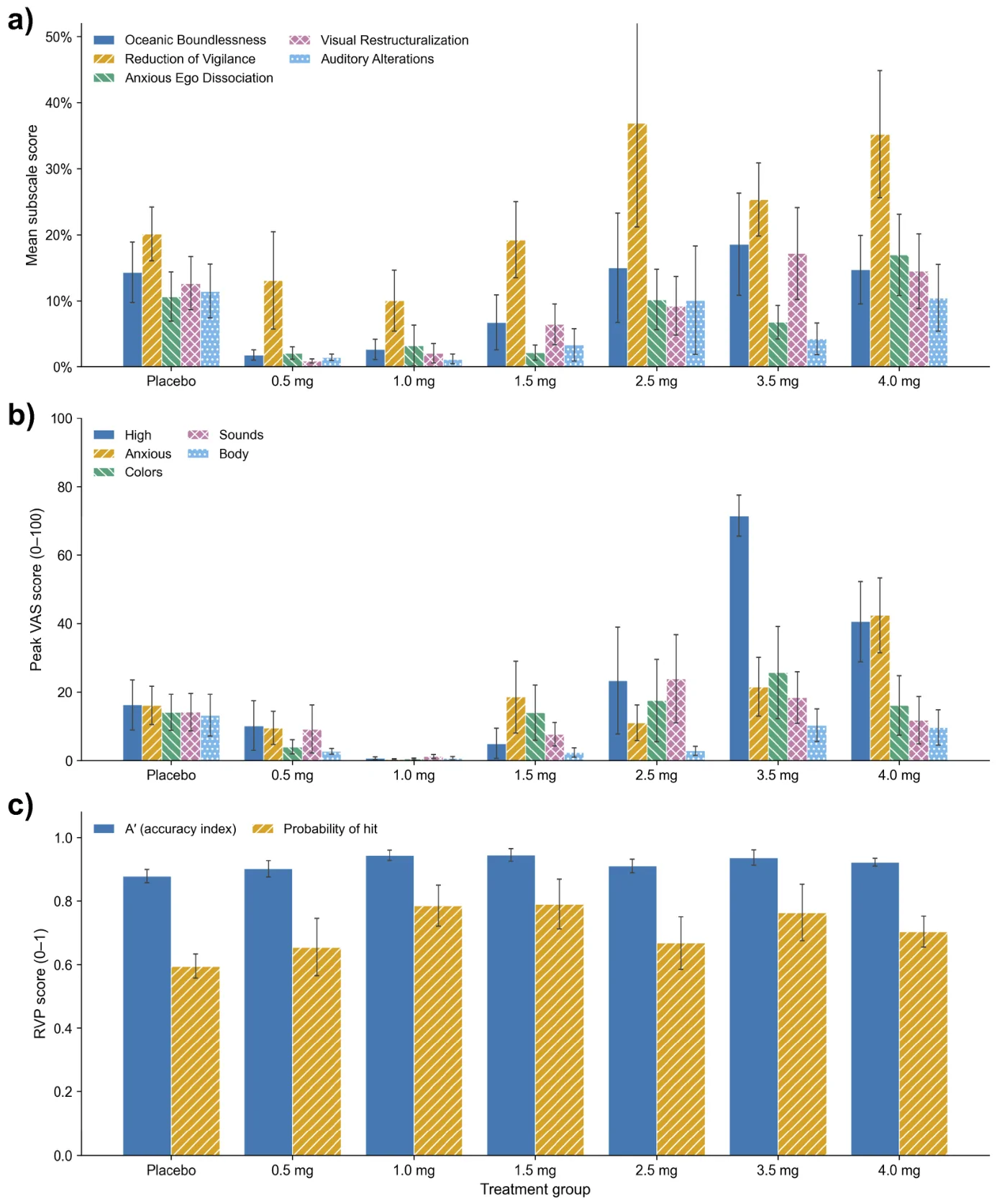
Influence of low-dose psilocybin on subjective experiences and visual attention. The histograms illustrate data (mean ± SEM) from altered states, peak perceptual effects, and sustained attention outcome measures. (**a**) 5D-ASC altered-states subscale scores (percentage of scale maximum) at 6 h post-dose. (**b**) Bowdle VAS peak (E_max_) ratings for the “High” and “Anxious” items and three perceptual-distortion items (Colors, Sounds, Body). (**c**) Rapid Visual Information Processing (RVP) A′ sensitivity index and probability of hit.

Dose-level summaries of the altered-states, perceptual, and attentional measures are shown in Figure 5. Scores on all five 5D-ASC dimensions were relatively low (<40%) across psilocybin doses up to 4.0 mg, indicating minimal alterations of consciousness (Figure 5a). The profile was dominated by the Reduction of Vigilance dimension reflecting drowsiness, which showed the highest scores (placebo ≈ 20%; 2.5 mg ≈ 37%; 4.0 mg ≈ 35%), whereas Oceanic Boundlessness (placebo ≈ 14%; 3.5 mg ≈ 19%), Anxious Ego Dissolution, Visionary Restructuralization, and Auditory Alterations all remained ≤19%. Dose trends were weak and non-significant (e.g., Oceanic Boundlessness ρ = 0.09, *p* = 0.53; Reduction of Vigilance ρ = 0.20, *p* = 0.13), and placebo scores were comparable to the active doses on several dimensions, consistent with non-specific or expectancy-related reporting rather than a psychedelic alteration of consciousness. The Reduction of Vigilance signal is most parsimoniously interpreted as mild drowsiness. Peak Bowdle perceptual ratings reinforced this interpretation (Figure 5b). The “High” item rose steeply at 3.5 and 4.0 mg, whereas items indexing overt perceptual distortions remained low across all doses. Sustained attention was preserved (Figure 5c). The mean RVP A′ remained high (≈ 0.88–0.95) across all groups, including the highest doses, with no dose-related decrement, and the probability of hit showed no systematic dose effect.

### 2.5. Psilocybin Dose–Response and Total Exposure of Subjective Effects Across Low Oral Doses

**Figure 6:**
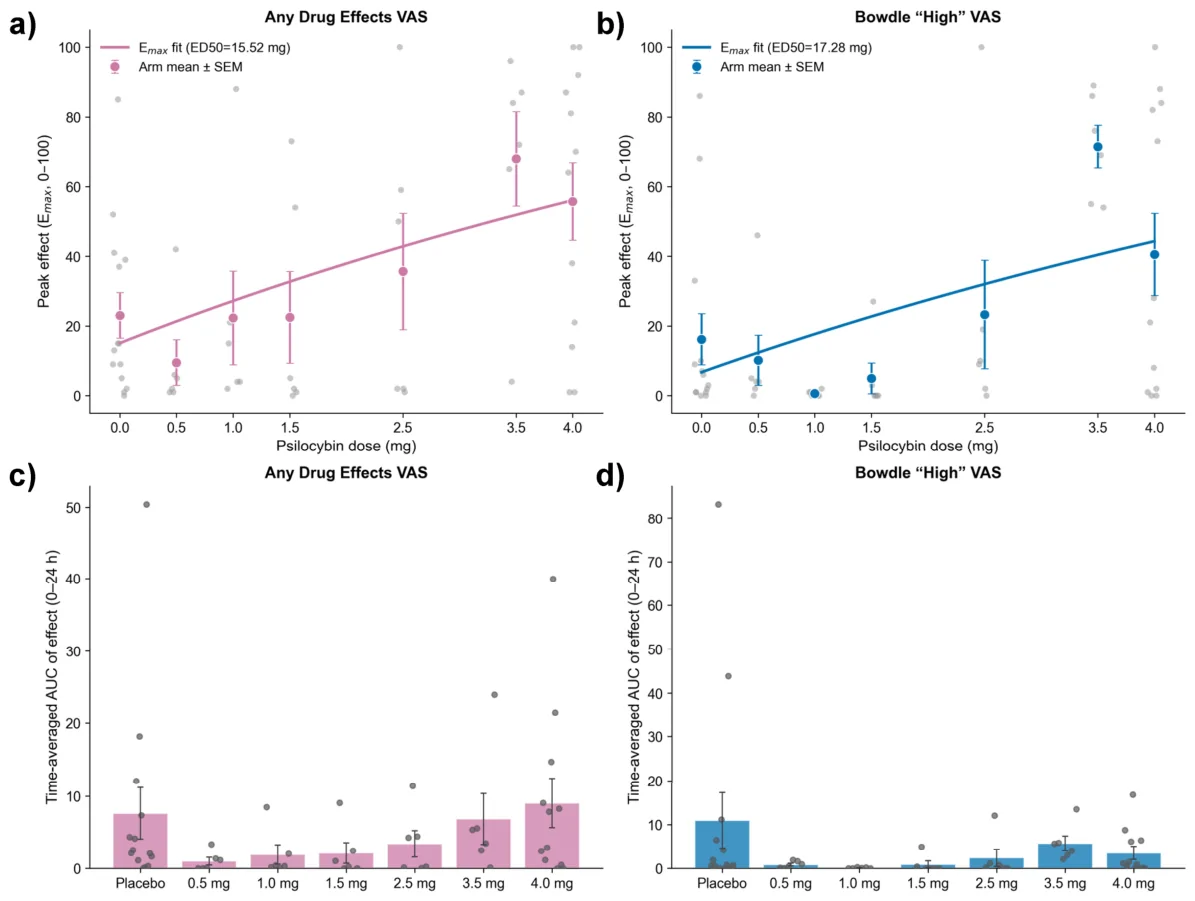
Dose response and total exposure of subjective effects experienced following administration of low-dose psilocybin. Peak (E_max_) Any Drug Effects (**a**) and Bowdle “High” VAS (**b**) for each participant (grey dots) with cohort mean ± SEM (colored dots) and a fitted three-parameter E_max_ model (colored line). Placebo is plotted at 0 mg. The Spearman ρ between dose and peak effect for Any Drug Effect was 0.34 (*p* = 0.01) and 0.30 (*p* = 0.03) for Bowdle “High” VAS scores. The histograms show the time-averaged area under the curve (AUC) of effects (0–24 h) by dose (mean ± SEM; grey dots represent individual data) for Any Drug Effects (**c**) and Bowdle “High” VAS (**d**). The Spearman ρ between dose and time-averaged area under the effect curve for Any Drug Effects was 0.14 (*p* = 0.29) and 0.09 (*p* = 0.49) for the Bowdle “High” VAS scores.

To quantify the dose relationship across time, we summarized each participant’s peak (E_max_) Any Drug Effects and Bowdle “High” rating as a function of dose (Figure 6a,b). Peak ratings increased monotonically with dose for both measures (Any Drug Effects: Spearman ρ = 0.34, *p* = 0.010; Bowdle “High”: ρ = 0.30, *p* = 0.025). Mean peak Any Drug Effects scores rose from 36 at 2.5 mg to 68 at 3.5 mg, and mean peak “High” scores from 23 to 72, respectively, compared to a placebo Any Drug Effect mean of 23 and mean “High” score of 16. Fitted E_max_ curves characterized a monotonic, non-saturating dose-response across the 0.0 to 4.0 mg range. Because the response did not plateau within the administered doses, the fitted ED_50_ was modeled beyond our observed data. Consequently, this model should be regarded strictly as a monotonic dose-response characterization for the sub-hallucinogenic window rather than a definitive E_max_ projection or absolute estimate of potency. Total drug-effect exposure, indexed by the time-averaged AUC of effect over 0–24 h, showed only a weak, non-significant dose relationship (Any Drug Effects ρ = 0.14, *p* = 0.29; Bowdle “High” ρ = 0.09, *p* = 0.49; Figure 6c,d), reflecting the brief, early time course of the subjective effect together with several placebo participants, who reported sustained low-level effects.

### 2.6. Influence of Low-Dose Psilocybin on Acute Cognitive Performance

**Figure 7:**
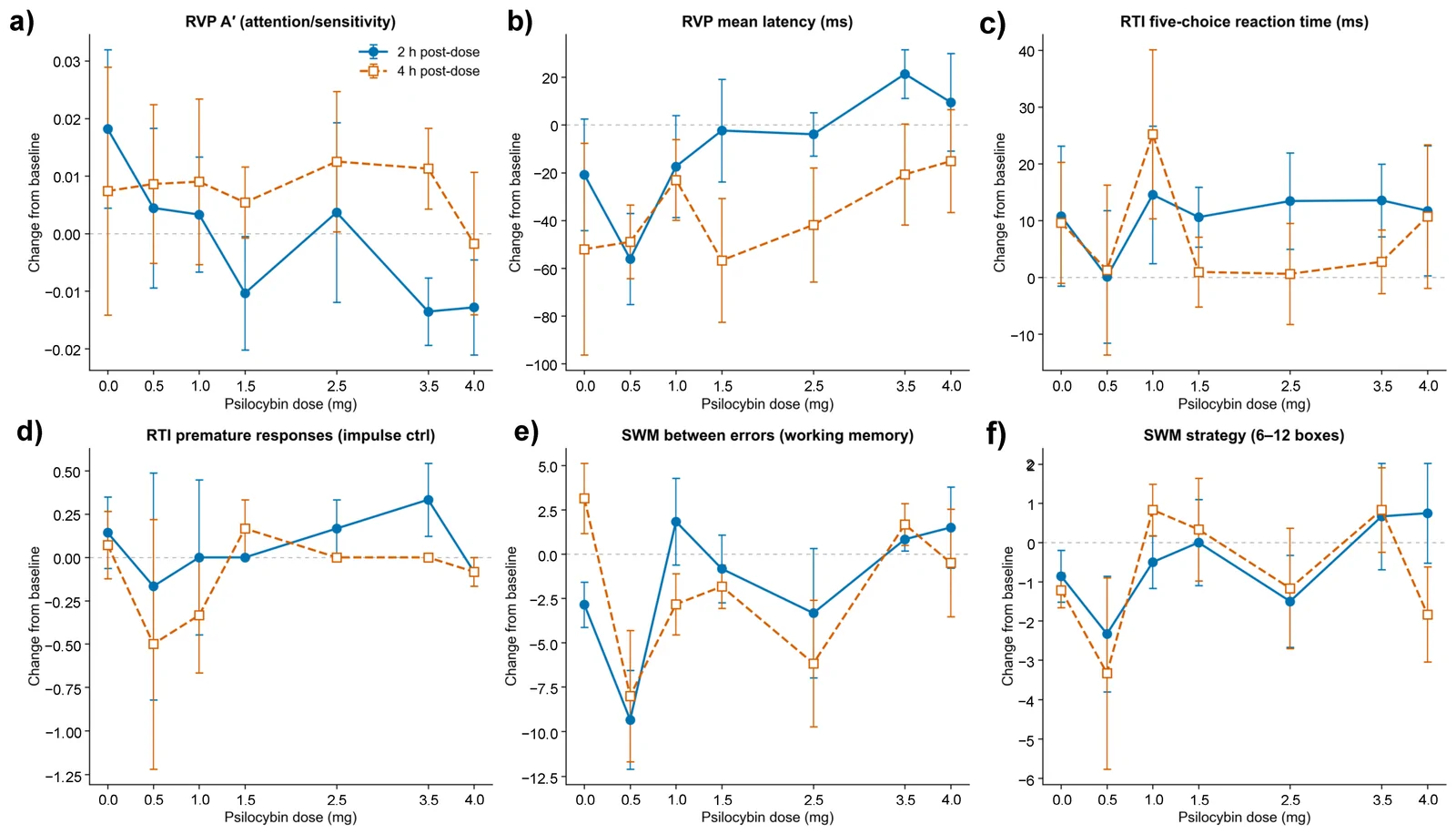
Influence of low-dose psilocybin on acute cognitive performance. The line plots illustrate change from baseline at 2 h (blue) and 4 h (orange) post-dose (mean ± SEM) for (**a**) RVP A′ (attention/sensitivity), (**b**) RVP mean latency, (**c**) RTI five-choice reaction time, (**d**) RTI premature responses (impulse control), (**e**) SWM between-search errors (working memory), and (**f**) SWM strategy.

Cognitive performance was preserved across the dose range, with no evidence of dose-dependent impairment (Figure 7). Several cognitive indices improved modestly over the session irrespective of treatment, consistent with task practice rather than a drug effect. The pooled RVP mean latency was faster at 4 h (−36.7 ms, *p* = 0.006), and SWM strategy improved at 4 h (change −0.96, *p* = 0.043). Where dose-related signals appeared, they were small and clustered around the 2 h exposure peak. Here we observed that RVP A′ slightly decreased at 2 h with dose (ρ = −0.28, *p* = 0.038), and the practice-related reduction in SWM between-search errors was attenuated at higher doses (ρ = +0.31, *p* = 0.020). The RTI five-choice reaction time was marginally slower at 2 h across participants (+10.8 ms, *p* = 0.016) and recovered by 4 h. Premature responses (impulse control) showed no consistent dose- or time-related changes. By 4 h, most indices had returned toward baseline (Figure 7), paralleling the falling psilocin plasma concentrations (Figure 2). Overall, performance on every task remained at or near baseline across the dose range. These data combined with subjective experiences indicate that the low doses of psilocybin engage cognitive affective systems, but in a manner where participants can complete attention, working-memory, and reaction-time tasks normally in the absence of distorted perceptions, altered sensations, or any dose-dependent deficits across the ranges examined.

### 2.7. Influence of Low-Dose Psilocybin on Psychophysiological Arousal Measured by Pupillometry

**Figure 8:**
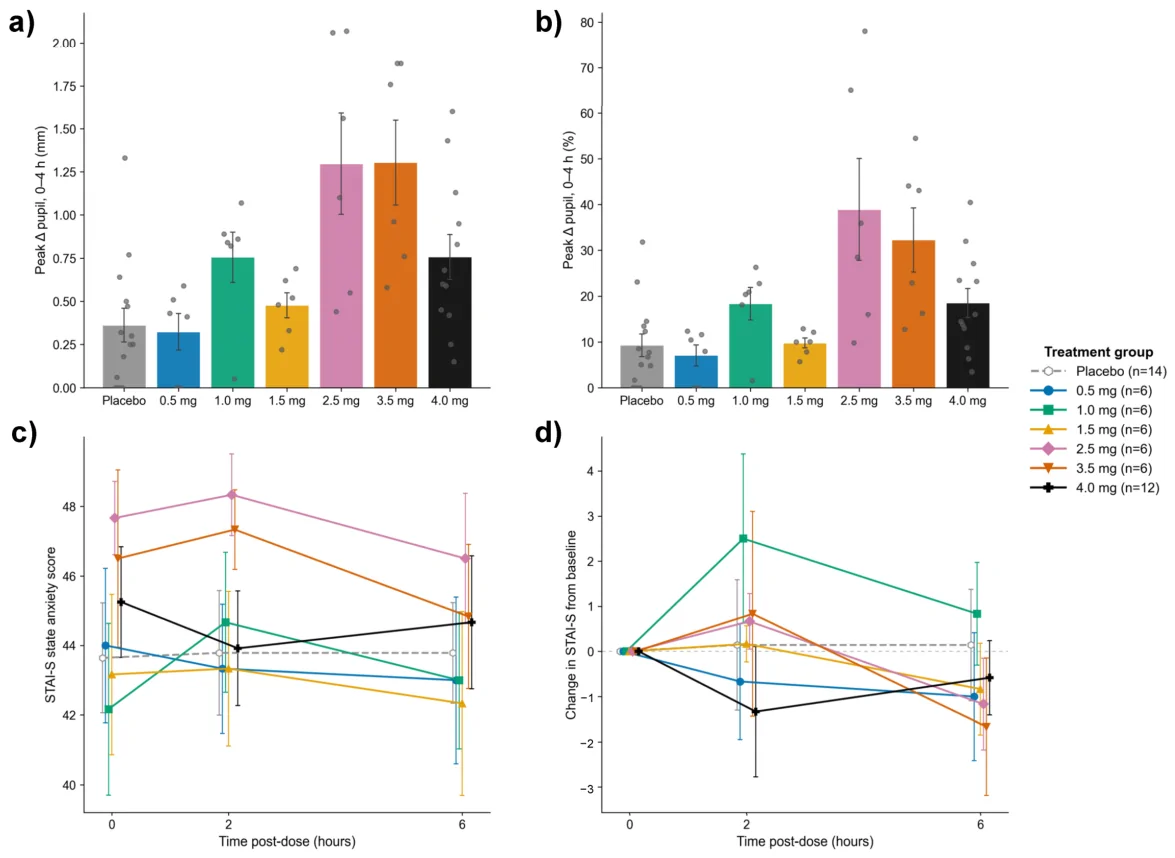
Effects of low-dose psilocybin on psychophysiological arousal and state anxiety. The histograms (**a**,**b**) illustrate peak pupil dilation within 0–4 h post-dose by dose, expressed in mm (**a**) and percent (**b**) change from baseline (mean ± SEM; grey dots show individual data). The line plots (**c**,**d**) illustrate state anxiety scores (STAI-State) over time by dose as absolute scores (**c**) and as change from each participant’s pre-dose baseline (**d**) shown as cohort means ± SEM.

Psilocybin produced a clear dose-dependent pupil dilation (Figure 8a,b). Pupil diameter increased within 0.5 to 1 h of dosing and was largest at the 2.5 and 3.5 mg doses. The mean dilation was significant relative to baseline at 1 h for 3.5 mg (+1.06 mm, *p* = 0.009) and 4.0 mg (+0.59 mm, *p* = 0.001) and at 2 h for 2.5 mg (+1.01 mm, *p* = 0.019), whereas placebo showed no increase at any time point (Figure 8a). Peak dilation within the first 4 h rose across most of the dose range (placebo +0.31 mm/+8%; 1.0 mg +0.75 mm/+18%; 2.5 mg +1.30 mm/+39%; 3.5 mg +1.30 mm/+32%; 4.0 mg +0.76 mm/+19%) and was correlated with dose when analyzed as raw change in mm from baseline (Spearman ρ = 0.46, *p* = 0.0004; Figure 8a), as well as percent change from baseline (ρ = 0.46, *p* = 0.0003; Figure 8b). Peak dilation was greater for psilocybin ≥2.5 mg than for placebo (Mann–Whitney *p* = 0.0003), and the maximum dilation likewise increased with dose (ρ = 0.37, *p* = 0.005), with the earliest time-to-peak at the higher doses. Collectively the data demonstrate a dose-dependent engagement of psychophysiological arousal produced by orally administered low-dose psilocybin, which occurs in absence of sensory/perceptual distortions or cognitive impairment.

### 2.8. Acute Effects of Low-Dose Oral Psilocybin on State Anxiety

High dose psilocybin has been reported to acutely increase anxiety even in healthy volunteers. Here we observed that state anxiety did not increase with psilocybin at any dose and, at the higher doses, produced a slight reduction (Figure 8c,d). STAI state scores showed participants had mild-to-moderate baseline levels of anxiety (group means 42–48 on the 20–80 scale). When examined as a change from each participant’s own pre-dose baseline, changes were small at doses >2.5 mg by 6 h (3.5 mg −1.7, 2.5 mg −1.2, 4.0 mg −0.6 points, versus +0.1 for placebo; pooled ≥3.5 mg −0.9 points, *p* = 0.21), with no significant effects on the total score. At the item level, however, several state items shifted in the anxiolytic direction with increasing dose by 6 h. For example, ratings of feeling “content” (Spearman ρ = +0.35, *p* = 0.009) and “secure” (ρ = +0.30, *p* = 0.023) increased, and feeling “frightened” (ρ = −0.26, *p* = 0.049) decreased. Trait anxiety was unaffected. These data revealed a consistent pattern of small reductions in state anxiety, together with increased feelings of security and contentment appearing at the higher, pharmacologically active doses. These observations in a healthy population are not taken to be predictive of clinical responses that may occur in patients suffering from anxiety disorders.

### 2.9. Effectiveness of Treatment Masking

When asked which treatment participants believed they had received, the largest single group of participants (23 of 56) responded that they did not know. Accuracy increased with dose, with more participants identifying their treatment as “certainly” or “probably” psychoactive at doses ≥2.5 mg. Among placebo recipients, only 3 of 14 incorrectly judged that they had received an active psychoactive drug. These findings indicate that the active-drug masking procedure was effective, particularly at the lower doses.

### 2.10. Acute Safety and Tolerability of Orally Administered Low-Dose Psilocybin

There were no deaths, no other serious adverse events, and no discontinuations due to treatment-emergent adverse events (TEAEs). All TEAEs were mild in intensity and considered related to study drug. The most frequently reported TEAE was somnolence, occurring across most psilocybin doses and placebo (Table 2). Euphoric mood (verbatim term: ‘feeling high’) was reported by 3 participants at 3.5 and 4.0 mg doses only. A single participant reported experiencing an auditory disturbance after receiving 4.0 mg, which was initially recorded as a mild hallucination TEAE. Review of the source documentation, however, revealed that the participant experienced ‘auditory disturbances and denied any visual disturbances, stating that he was unable to focus and gather his thoughts as he was having random thoughts in his mind and getting flashbacks of old memories.’ Upon further review, the reported experience was more consistent with a ‘thought disturbance’ or distractable attention rather than a hallucination. In fact, this clinical clarification and context explain why subjective Hallucination VAS scores remained low and did not differ across doses or placebo (Figure 4). The incidence of TEAEs was generally lower at doses ≤1.5 mg, higher at doses >1.5 mg (except the 3.5 mg group), and intermediate following placebo. Mean clinical laboratory, vital sign, and ECG values remained within normal ranges, with no clinically significant findings, and no participant exhibited suicidal ideation or behavior on the C-SSRS. Treatment-emergent adverse events are summarized in Table 2.

## 3. Discussion

In this Phase 1 single-ascending-dose study, oral psilocybin administered at doses of 0.5 to 4.0 mg was well tolerated in healthy adults. No serious adverse events or discontinuations occurred, all treatment-emergent adverse events were mild, and no clinically significant changes were observed in laboratory parameters, vital signs, electrocardiograms, or suicidality assessments. This safety profile is consistent with prior reports of sub-hallucinogenic psilocybin dosing [5,6,12,23,24]. Under blinded and controlled conditions, oral psilocybin doses in the 2.5–4.0 mg range produced measurable, dose-graded pharmacodynamic responses including objective pupillary dilation and a consistent pattern of reduced state anxiety that are not attributable to expectancy alone. Participant-reported drug effects on the Any Drug Effects and Bowdle “High” visual analog scales (VAS) increased with dose and plasma psilocin exposure, becoming distinguishable from placebo at doses ≥2.5 mg and reaching their greatest magnitude at 3.5 and 4.0 mg, with an orderly, monotonic dose–response relationship for peak effect (Spearman ρ ≈ 0.30–0.34). This perceptible effect was not accompanied by hallucinatory activity that could be reliably distinguished from placebo (Figure 4). Scores on the 5D-ASC indicated only minimal alteration of consciousness, and Bowdle perceptual-distortion items (colors, sounds, body) remained low across the dose range despite increasing “High” ratings (Figure 5). These findings provide direct evidence in humans that the perceptible pharmacology of psilocybin can be dissociated from its hallucinogenic effects at low doses, addressing a central and previously unresolved translational question in the field.

A relevant safety outcome for an outpatient therapy is that cognition was preserved across the entire dose range. Attention (RVP A′), vigilance (RVP and RTI latencies), working memory (SWM errors and strategy), and impulse control (RTI premature responses) showed no dose-dependent decrements (Figure 7). The modest changes observed were largely practice-related and present in placebo. Performance across all cognitive tasks remained at or near baseline throughout the assessment period, indicating a pharmacologically active effect that did not manifest as measurable cognitive impairment, even at the highest doses evaluated. This absence of cognitive impairment across the dose range is consistent with, albeit not sufficient on its own to establish, the feasibility of unsupervised, outpatient administration.

Psilocybin produced a clear, dose-graded pupillary dilation that paralleled plasma psilocin and the subjective-effect time course (peak ~1 h; Figure 8a,b). Because pupil diameter under constant luminance is a sensitive peripheral index of autonomic nervous system activity and sympathetic tone, this dilation provides an objective biomarker confirming measurable autonomic and arousal-system engagement even at these low, sub-hallucinogenic doses. The convergence of a measurable physiological signal with intact cognition indicates that the doses studied are pharmacologically active without producing overt functional impairment. Pharmacokinetic data also support this interpretation. Psilocin appeared rapidly and was relatively short-lived (median terminal half-life ~1.6–2.5 h), and exposure increased predictably with dose. This pharmacokinetic profile of rapid onset, brief duration, and dose-proportional exposure is consistent with a pharmacodynamic window suitable for outpatient dosing paradigms, where a short and predictable window of drug effect is advantageous. The absence of an acute increase in state anxiety at any dose and indeed the slight reduction in STAI-State scores (Figure 8d) at the upper, pharmacologically active doses, accompanied by dose-dependent increases in feeling “content” (ρ = +0.35) and “secure” (ρ = +0.30) and a decrease in feeling “frightened” (ρ = −0.26) by 6 h is an encouraging on-target signal for an anxiety indication. However, this mild anxiolytic signal in healthy individuals is not taken to be predictive of any clinical efficacy. Future studies will be required to specifically evaluate safety, tolerability, and therapeutic efficacy in clinical populations.

### 3.1. Rationale for Advancing 3 mg Psilocybin in Clinical Investigations

Identification of a low, sub-hallucinogenic dose threshold addresses a gap between widespread naturalistic use of low-dose psilocybin and the availability of controlled clinical data to inform its development. Survey and naturalistic studies report that low-dose psilocybin use is associated with reduced state anxiety and depressive symptoms [12,13,14,25], and psilocybin has been characterized across a range of doses as having a favorable toxicity profile and low potential for dependence or abuse [26,27,28,29,30,31]. These observational findings are informative but are inherently subject to expectancy bias and selection effects, limitations that necessitate the controlled pharmacological characterization provided by the present study.

The present data indicate that doses up to 4.0 mg were tolerated without cognitive or psychomotor impairment, and that doses between 2.5 and 3.5 mg constituted a window of measurable pharmacological engagement, evidenced by dose-dependent pupillary dilation, in the absence of significant perceptual alteration or hallucinatory activity. Doses below 2.5 mg produced subjective effects that were largely indistinguishable from placebo, suggesting insufficient receptor engagement to produce a reliably perceptible effect. The 2.5 mg dose approximated the lower boundary of perceptibility, with “High” VAS ratings comparable to placebo but a measurable pupillary response. At 3.5 mg and 4.0 mg, subjective drug effects were clearly present, with the principal signals of perceptual or mood alteration limited to euphoric mood (reported verbatim as “feeling high”). A single hallucination-coded adverse event, characterized in source documentation as transient disorganized thought rather than a classical perceptual hallucination, occurred at 4.0 mg. On this basis, a 3 mg dose is estimated to lie between the 2.5 mg sub-perceptual threshold and the 3.5 mg dose at which the earliest perceptual effects were observed and is therefore proposed as a candidate dose positioned to be pharmacologically active while remaining below the threshold for perceptual or mood-altering effects.

This estimate is consistent with preliminary clinical data from an open-label trial in patients with advanced illness and severe psychological distress, in which oral psilocybin titrated from 1 to 3 mg daily was well tolerated, produced no reported psychedelic experience, and was associated with improvement in depression, anxiety, and demoralization measures, with the greatest reported benefit occurring at the 3 mg dose in the majority of responders [16]. The concordance between this independent clinical observation and the pharmacological threshold identified in the present study provides converging, though not confirmatory, support for advancing a 3 mg dose into controlled outpatient investigation, including ongoing trials in generalized anxiety disorder (GAD).

### 3.2. Putative Therapeutic Mechanisms of Action for Low-Dose Psilocybin

The safety and pharmacodynamic profile observed in this study is consistent with a model in which sub-hallucinogenic psilocybin engages central serotonergic and neurotrophic signaling pathways without producing the cortical receptor engagement associated with classical hallucinatory experience. Preclinical evidence indicates that psilocin’s high lipophilicity permits diffusion across the neuronal membrane to engage an intracellular pool of 5-HT2A receptors, a mechanism proposed to underlie sustained intracellular signaling and structural neuroplasticity independent of surface-receptor-mediated perceptual effects [21,32,33]. Pharmacological data in rodent models support a dissociation between these pathways, as follows: 5-HT2A/C receptor antagonism attenuates hallucinogenic behavioral markers without blocking synaptic plasticity in depression models [34], suggesting that neuroplastic effects may be substantially mediated by non-classical signaling mechanisms, including direct action on BDNF/TrkB signaling [19,20,34].

Additional preclinical work indicates that psilocin may act as a positive allosteric modulator at the TrkB receptor transmembrane domain, with reported affinity substantially exceeding that of conventional antidepressants [20]. At the low plasma concentrations achieved in the present study, this mechanism could plausibly support BDNF-dependent signaling without engaging the surface 5-HT2A activity associated with hallucinogenic effects at higher exposures [20] and is consistent with reported increases in presynaptic vesicle glycoprotein density in prefrontal and hippocampal regions following single-dose psilocybin in animal models [33]. Agonism at 5-HT1A receptors represents an additional candidate mechanism relevant to the anxiolytic signal observed in this study [21]. These mechanistic hypotheses remain to be tested directly in humans. The present data do not establish mechanism and should be interpreted as providing a plausible biological framework consistent with the observed pharmacodynamic profile rather than evidence of a specific molecular pathway.

### 3.3. Bioethical Considerations

Low-dose or subperceptual psilocybin use by the general public has outpaced the controlled safety data needed to evaluate it, creating a distinct regulatory and ethical challenge from the high-dose, supervised psychedelic model. Accordingly, while this single-ascending-dose study addresses an important evidence gap, its design and implementation required several ethical safeguards. Administering a psychoactive controlled substance to healthy adults with no prospect of direct therapeutic benefit requires justification beyond standard Phase 1 practice. The risk–benefit rationale here rested on three design features intended to minimize harm while preserving scientific validity, as follows: (1) a conservative, pharmacokinetically-informed starting dose (0.5 mg) selected specifically to remain below the established perceptual threshold [3,35]; (2) a stepwise single-ascending-dose design with mandatory Drug Safety Review Committee reviews between cohorts, empowered to halt escalation on pre-specified stopping rules (serious drug-related TEAEs in ≥2 participants); and (3) continuous inpatient monitoring for 24 h post-dose during the highest-risk window.

A methodologically distinctive and ethically consequential feature of this trial was the active-drug masking procedure, in which participants were told during consent that they might receive one of five pharmacologically distinct agents (niacin, alprazolam, ibuprofen, psilocybin, or methylphenidate) or placebo, when in fact only psilocybin or inert placebo were ever administered. This constitutes an incomplete disclosure of the trial’s true design, which departs from a strict standard of full transparency in informed consent. This departure was deliberate and, we argue, ethically defensible on methodological grounds specific to psychedelic research. Psilocybin’s characteristic subjective effects (e.g., altered perception) make standard blinding exceptionally difficult to preserve when participants and unblinded staff can often infer treatment assignment from drug effects alone, undermining the validity of blinded safety and pharmacodynamic assessments and reintroducing expectancy bias, a well-documented confound in this literature [12,13]. Active-drug masking is an established mitigation strategy in psychedelic trials. Three safeguards were used to keep this within ethically acceptable limits, as follows: (1) participants were informed prospectively and explicitly, during consent, that the true identity of their treatment could be masked among several possibilities; (2) all five masking candidates are approved, well-characterized compounds with established safety profiles, so no participant was exposed to undisclosed risk; and (3) participants were debriefed and asked to guess their treatment prior to discharge, both to assess masking effectiveness and to close the consent loop before leaving the study site. We consider this an implementation of “authorized deception” rather than concealment, consistent with precedent in the psychedelic clinical trials literature, though we acknowledge this remains a point of ongoing methodological and ethical debate in the field and note that fully transparent alternatives (e.g., simple double-blind placebo control) remain preferable where blinding integrity can otherwise be maintained.

Because participants were explicitly told psychoactive effects were possible, the study incorporated the C-SSRS at multiple timepoints and real-time clinical staff observation to detect any emergent distress, self-harm ideation, or adverse psychological reaction independent of expectancy state. No participant exhibited suicidal ideation or behavior during the study, and all AEs were clinically characterized as transient and non-distressing. With respect to data analyses and iterpretations, all pharmacodynamic and exploratory analyses conducted beyond the pre-specified descriptive safety and PK analyses are reported as exploratory and hypothesis-generating, with uncorrected *p*-values (Section 2.8). We disclose this explicitly to avoid overstating the confirmatory strength of dose–response and anxiolytic signals that informed the dose-selection rationale in Section 4.1, given the downstream implication that these data support advancing a specific dose (3 mg) into further trials.

### 3.4. Limitations and Future Directions

Several limitations warrant consideration. As a first-in-program Phase 1 study, the trial design was exploratory, with a modest overall sample size, small per-cohort group sizes, and pre-specified descriptive rather than inferential statistical analyses. Cognitive assessments were conducted at 0, 2, and 4 h post-dose rather than at the approximate 1 h pharmacodynamic peak, and baseline cognitive performance in this healthy sample was near ceiling. Both factors may have reduced sensitivity to detect subtle acute cognitive change. The fitted Emax model did not reach a plateau within the administered dose range, and the derived ED_50_ should therefore be regarded as an extrapolation rather than an empirically anchored estimate. The study population consisted of healthy adults rather than patients with GAD, limiting direct inference to the intended clinical population, particularly with respect to anxiolytic signals, given the constrained baseline anxiety range in this sample. Subjective and altered-states measures relied on self-report and are susceptible to expectancy effects, notwithstanding the active-drug masking procedure, which appeared effective at reducing expectancy-driven bias, particularly at lower doses. Future work should evaluate repeated low-dose administration across extended timeframes and incorporate additional objective physiological measures, such as electroencephalography and heart-rate variability, to further characterize central and autonomic correlates of this dose range.

## 4. Materials and Methods

### 4.1. Study Design and Regulatory Oversight

This was a Phase 1, randomized, double-blind, placebo-controlled, single-ascending- dose (SAD) study conducted in healthy adult men and women at a single clinical research site (BioPharma Services Inc., Toronto, ON, Canada). The study was conducted in accordance with the Declaration of Helsinki and International Council for Harmonization Good Clinical Practice guidelines. The Clinical Trial Application for psilocybin (control number 254766; Health Canada file HC6-24-c254766) was reviewed by the Therapeutic Products Directorate of Health Canada, and a No Objection Letter for the protocol was issued on 13 August 2021. Independent ethics approval was granted by Advarra (Aurora, ON, Canada) on 1 November 2021 for the protocol #2626 prior to any participant recruitment or enrollment. Because psilocybin is a controlled substance in Canada, the trial was additionally conducted under the applicable exemption granted under the Canadian Controlled Drugs and Substances Act. Written informed consent was obtained from all individual participants included in the study. Study enrollment began on 3 November 2021, treatment began on 5 November 2021, and study treatment and follow-up were completed 12 March 2022. At the time and place the study was conducted, there was no statutory requirement for the registration of Phase 1 trials. We therefore did not prospectively register this study. However, to ensure data sharing and public transparency, the trial was retrospectively registered following study completion with ClinicalTrials.gov (#NCT07710027; https://clinicaltrials.gov/study/NCT07710027 (accessed on 9 September 2026)) on 17 July 2026. The CONSORT diagram for the trial and dose-escalation study flow design is summarized in Figure 1.

The study comprised three visits, as follows: an outpatient screening visit (Visit 1) within 28 days of admission; a 3-day (2-night) inpatient treatment phase (Visit 2; Day 1 to Day 2); and an outpatient follow-up visit (Visit 3) 7 ± 2 days after dosing. Safety and pharmacodynamic data were collected for up to 24 h post-dose during the inpatient phase.

### 4.2. Participants

Eligible participants were healthy adults aged 18 to 55 years with a body mass index of 18.0 to 34.0 kg/m^2^ (minimum weight 50 kg), resting blood pressure within protocol-defined limits (systolic 95–140 mmHg; diastolic 55–90 mmHg), and non-smoker status for at least 6 months. Key exclusion criteria included any clinically significant cardiac, neurologic, hepatic, psychiatric or other systemic disease; a personal or immediate-family history of schizophrenia, bipolar disorder, or other psychotic disorders; a current or recent (within 5 years) history of major depression, obsessive–compulsive disorder, panic disorder, generalized or social anxiety disorder, or an eating disorder; any history of suicidal ideation or behavior per the Columbia-Suicide Severity Rating Scale (C-SSRS); use of perception-altering substances (e.g., LSD, MDMA or psilocybin) for non-therapeutic purposes within the prior 5 years or on five or more lifetime occasions; and a negative drug screen urinalysis or breath alcohol test at admission.

### 4.3. Randomization, Blinding, and Masking of Treatment

Within each cohort of 8 participants, 6 were randomized to a single oral dose of psilocybin and 2 to matching placebo (3:1 ratio), with a minimum of one participant of each sex assigned to psilocybin. Randomization codes were generated by an unblinded statistician using software-generated procedures (SAS 9.4) and held by an unblinded pharmacist. Participants, investigators, site staff, and the bioanalytical laboratory were otherwise blinded to treatment assignment.

To minimize expectancy effects, a recognized confound in psychedelic trials [12], an active-drug masking procedure was used [13]. During consent, participants were informed verbally and in writing that they might receive placebo or any one of five drugs spanning several pharmacological classes (niacin, alprazolam, ibuprofen, psilocybin, or methylphenidate) while only psilocybin or matching placebo were administered in a masking procedure. Prior to discharge, participants were asked which treatment they believed they had received, to assess the effectiveness of masking.

### 4.4. Dosing, Dose Escalation, and Threshold-Dose Definition

We used cGMP manufactured psilocybin (Dalton Pharma Services, Toronto, ON, Canada) administered orally as an aqueous solution containing 0.2% sucralose as a taste-masking agent. The placebo was produced with an identical formulation and identical container-closure components with the omission of psilocybin, so that active and placebo treatments were indistinguishable in appearance, volume, and taste. The starting dose was 0.5 mg, selected based on pharmacokinetic and receptor-occupancy data indicating that doses of approximately 2 mg or less would yield plasma psilocin concentrations below the perceptible threshold of about 4 to 6 ng/mL [3,35]. The volume of sucralose was 3 mL for the 0.5 mg dose and matching placebo and 6 mL for all other doses and matched placebo administrations. The protocol permitted dose levels up to a maximum of 5 mg.

Following completion of each cohort, a Drug Safety Review Committee (DSRC) reviewed blinded safety and pharmacodynamic data through 24 h post-dose and determined the next dose level (Figure 1b). Escalation was to be stopped if any drug-related serious adverse event occurred, if two or more participants experienced moderate drug-related neuropsychiatric adverse events, or if the DSRC concluded that a dose exceeding a non-psychoactive threshold had been reached. The threshold dose was defined a priori as the dose immediately preceding that at which escalation was halted. The actual escalation sequence used in the trial was 0.5, 1.0, 1.5, 2.5, 3.5, and 4.0 mg; the planned 2.0 and 3.0 mg levels were not administered, and the 4.0 mg level was repeated in an additional cohort yielding n = 12 participants at 4.0 mg.

### 4.5. Outcomes and Safety Assessments

The primary outcome was safety and tolerability, assessed as the incidence, severity, and relationship to study drug of treatment-emergent adverse events, together with vital signs, 12-lead ECG, clinical laboratory tests, physical examination, and C-SSRS. Secondary outcomes were the pharmacodynamic and pharmacokinetic effects of single ascending doses. Assessment of perceived treatment assignment (expectancy) was a pre-specified exploratory outcome. Safety was assessed through adverse event monitoring, vital signs, 12-lead electrocardiograms (ECGs), clinical laboratory tests, physical examinations, concomitant medication review, and administration of the C-SSRS. Adverse events were coded using the Medical Dictionary for Regulatory Activities (MedDRA, version 24.0) and graded for severity and relationship to study drug by the investigator.

### 4.6. Pharmacokinetic Assessments

Venous blood samples for plasma psilocin concentrations were collected pre-dose and at 0.25, 0.5, 1, 1.5, 2, 3, 4, 6, 8, 10, 12, 18, and 24 h (h) post-dose and analyzed using a validated bioanalytical method. The pharmacokinetic parameters maximum observed concentration (C_max_), time to C_max_ (T_max_), area under the concentration–time curve to the last measurable concentration (AUC_last_) and to infinity (AUC∞), as well as terminal elimination half-life (t½) were derived by non-compartmental analysis (Phoenix WinNonlin; Certara, Princeton, NJ, USA). Dose proportionality was assessed with a power model on natural-log-transformed C_max_, AUC_last_, and AUC∞, concluding proportionality if the 90% confidence interval for the slope fell entirely within 0.80 to 1.25.

### 4.7. Pharmacodynamic Assessments

Subjective effects were assessed using 100-point visual analog scales (VAS) for Alertness/Drowsiness, Agitation/Relaxation, Hallucinations, and Any Drug Effects; the Bowdle VAS (internal- and external-perception composites and individual items, including the “I felt high” item and perceptual-distortion items for colors, sounds, and body) [36]; and the Bond-Lader VAS [37]. Alterations of consciousness were assessed once, at 6 h post-dose, using the 5-Dimensional Altered States of Consciousness (5D-ASC) scale including Oceanic Boundlessness, Anxious Ego Dissolution, Visionary Restructuralization, Auditory Alterations, and Reduction of Vigilance [38]. Anxiety was assessed with the State-Trait Anxiety Inventory (STAI-State and STAI-Trait) at 0, 2, and 6 h [39]. Cognitive and psychomotor function was assessed with the CANTAB Reaction Time (RTI, five-choice), Rapid Visual Information Processing (RVP), and Spatial Working Memory (SWM) tasks at 0, 2, and 4 h post-dose (Cambridge Cognition Ltd., Toronto, ON, Canada). Reported indices were RVP A′ (target sensitivity), RVP probability of hit and response latency, RTI five-choice reaction time and premature responses (an index of impulse control), and SWM between-search errors (working memory) and strategy score. Pupil diameter (right eye) was measured by pupillometry pre-dose and at 0.5, 1, 2, 3, 4, 6, 8, 10, 12, and 24 h as an objective physiological measure of autonomic arousal.

### 4.8. Statistical Analysis

The sample size was based on precedent from comparable Phase 1 studies. The safety population comprised all participants who received study drug, and the pharmacokinetic population all psilocybin recipients with evaluable concentration data. Safety and pharmacodynamic data were summarized descriptively by dose level; no inferential between-group hypothesis testing was prespecified, consistent with the exploratory objectives of a first-in-program Phase 1 study. Pharmacokinetic parameters were summarized with arithmetic and geometric descriptive statistics and dose proportionality evaluated by the power model above.

Pharmacodynamic and exploratory analyses were performed in Python 3.9 (pandas, NumPy, SciPy, Matplotlib 3.11.0). Subjective-effect time courses are shown as arm means ± standard error of the mean (SEM) by dose (Figure 3). For each participant the peak (maximum post-dose) Any Drug Effects and Bowdle “High” rating (Emax) and the time-averaged area under the effect–time curve (AUC, 0–24 h) were computed; their dose relationships were summarized by Spearman rank correlation, and Emax additionally by a three-parameter Emax model (E = E_0_ + E_max_·D⁄(ED_50_ + D)) fitted by non-linear least squares (Figure 5). Pupil-diameter change from baseline was computed at each time point and as the peak change within 0–4 h; the dose relationship was tested by Spearman correlation, within-arm dilation against zero by one-sample t-test, and psilocybin ≥2.5 mg versus placebo by Mann–Whitney U test (Figure 7a,b). Cognitive change from baseline at 2 and 4 h was tested against zero (one-sample *t*-test) and for a dose relationship (Spearman; Figure 6). State anxiety (STAI-State) was summarized as absolute and change-from-baseline scores, with dose trends in the total and individual state-item scores assessed by Spearman correlation (Figure 7c,d). The pupillometry and cognitive analyses were conducted and are reported independently, reflecting their distinct purposes (an autonomic physiological readout versus task performance). No imputation was performed for missing data. All 56 randomized participants completed the study; pharmacokinetic evaluability was assessed on a case-by-case basis, yielding a pharmacokinetic population of 42 psilocybin recipients. Because all pharmacodynamic analyses were descriptive and exploratory, effect estimates are presented as group means ± SEM without confidence intervals, and *p*-values are descriptive and uncorrected.

## 5. Conclusions

In conclusion, single oral doses of psilocybin up to 4.0 mg were well tolerated in healthy adults and produced dose-related, perceptible subjective effects together with an objective, dose-graded pupillary response, in the absence of clinically meaningful altered states of consciousness, distorted perceptions, diminished cognitive abilities, or psychomotor impairment. These findings support the pharmacological separability of psilocybin’s perceptible effects from its hallucinogenic activity at low doses and identify a candidate dose range for further evaluation in outpatient clinical settings.

## Figures and Tables

**Table 1:** Participant demographics and baseline characteristics.

Characteristic	0.5 mg (n = 6)	1.0 mg (n = 6)	1.5 mg (n = 6)	2.5 mg (n = 6)	3.5 mg (n = 6)	4.0 mg (n = 12)	Placebo (n = 14)	Total (N = 56)
Age, years, mean (SD)	32.8 (9.5)	35.8 (11.4)	32.3 (6.7)	36.5 (12.3)	33.0 (9.7)	43.8 (8.6)	39.5 (10.2)	37.5 (10.2)
Sex, n (%)								
Male	3 (50.0)	2 (33.3)	3 (50.0)	3 (50.0)	2 (33.3)	7 (58.3)	8 (57.1)	28 (50.0)
Female	3 (50.0)	4 (66.7)	3 (50.0)	3 (50.0)	4 (66.7)	5 (41.7)	6 (42.9)	28 (50.0)
Race, n (%)								
Asian	4 (66.7)	0	0	1 (16.7)	0	3 (25.0)	4 (28.6)	12 (21.4)
Black	1 (16.7)	3 (50.0)	2 (33.3)	2 (33.3)	2 (33.3)	4 (33.3)	4 (28.6)	18 (32.1)
White	1 (16.7)	3 (50.0)	4 (66.7)	3 (50.0)	4 (66.7)	5 (41.7)	6 (42.9)	26 (46.4)
Ethnicity, n (%)								
Hispanic/Latino	1 (16.7)	2 (33.3)	2 (33.3)	3 (50.0)	1 (16.7)	1 (8.3)	1 (7.1)	11 (19.6)
Not Hispanic/Latino	5 (83.3)	4 (66.7)	4 (66.7)	3 (50.0)	5 (83.3)	11 (91.7)	13 (92.9)	45 (80.4)
BMI, kg/m^2^, mean (SD)	24.38 (3.04)	25.47 (4.89)	27.32 (3.68)	24.80 (3.35)	23.55 (1.50)	27.34 (3.23)	27.87 (4.53)	26.28 (3.86)

SD, standard deviation; BMI, body mass index. Percentages use the number of participants per treatment group as the denominator.

**Table 2:** Treatment-emergent adverse events by preferred term and dose (safety population).

Preferred Term, n (%)	0.5 mg (n = 6)	1.0 mg (n = 6)	1.5 mg (n = 6)	2.5 mg (n = 6)	3.5 mg (n = 6)	4.0 mg (n = 12)	Placebo (n = 14)	Total (N = 56)
Any TEAE	2 (33.3)	2 (33.3)	2 (33.3)	3 (50.0)	2 (33.3)	6 (50.0)	6 (42.9)	23 (41.1)
Somnolence	2 (33.3)	0	2 (33.3)	2 (33.3)	0	2 (16.7)	5 (35.7)	13 (23.2)
Dizziness	0	1 (16.7)	0	0	1 (16.7)	1 (8.3)	1 (7.1)	4 (7.1)
Headache	0	0	0	1 (16.7)	0	1 (8.3)	0	2 (3.6)
Euphoric mood	0	0	0	0	1 (16.7)	2 (16.7)	0	3 (5.4)
Nausea	0	1 (16.7)	0	1 (16.7)	0	0	0	2 (3.6)
Vision blurred	0	0	0	0	0	1 (8.3)	1 (7.1)	2 (3.6)
Hallucination	0	0	0	0	0	1 (8.3)	0	1 (1.8)
Hypervigilance	0	1 (16.7)	0	0	0	0	0	1 (1.8)
Dry eye	0	0	0	0	0	0	1 (7.1)	1 (1.8)
Fatigue	0	0	0	1 (16.7)	0	0	0	1 (1.8)
Sluggishness	0	0	0	0	0	1 (8.3)	0	1 (1.8)
Rash	0	0	0	0	0	0	1 (7.1)	1 (1.8)

TEAE, treatment-emergent adverse event. All events were mild in severity and considered related to study drug. Events were coded with MedDRA version 24.0; percentages use the number of participants per group as the denominator.

## Data Availability

The raw data supporting the conclusions of this article will be made available by the authors on request.
